# Evolutionary rate patterns of the Gibberellin pathway genes

**DOI:** 10.1186/1471-2148-9-206

**Published:** 2009-08-18

**Authors:** Yan-hua Yang, Fu-min Zhang, Song Ge

**Affiliations:** 1 State Key Laboratory of Systematic and Evolutionary Botany, Institute of Botany, Chinese Academy of Sciences, Beijing 100093, PR China; 2 Graduate University, Chinese Academy of Sciences, Beijing 100039, PR China

## Abstract

**Background:**

Analysis of molecular evolutionary patterns of different genes within metabolic pathways allows us to determine whether these genes are subject to equivalent evolutionary forces and how natural selection shapes the evolution of proteins in an interacting system. Although previous studies found that upstream genes in the pathway evolved more slowly than downstream genes, the correlation between evolutionary rate and position of the genes in metabolic pathways as well as its implications in molecular evolution are still less understood.

**Results:**

We sequenced and characterized 7 core structural genes of the gibberellin biosynthetic pathway from 8 representative species of the rice tribe (Oryzeae) to address alternative hypotheses regarding evolutionary rates and patterns of metabolic pathway genes. We have detected significant rate heterogeneity among 7 GA pathway genes for both synonymous and nonsynonymous sites. Such rate variation is mostly likely attributed to differences of selection intensity rather than differential mutation pressures on the genes. Unlike previous argument that downstream genes in metabolic pathways would evolve more slowly than upstream genes, the downstream genes in the GA pathway did not exhibited the elevated substitution rate and instead, the genes that encode either the enzyme at the branch point (GA20ox) or enzymes catalyzing multiple steps (KO, KAO and GA3ox) in the pathway had the lowest evolutionary rates due to strong purifying selection. Our branch and codon models failed to detect signature of positive selection for any lineage and codon of the GA pathway genes.

**Conclusion:**

This study suggests that significant heterogeneity of evolutionary rate of the GA pathway genes is mainly ascribed to differential constraint relaxation rather than the positive selection and supports the pathway flux theory that predicts that natural selection primarily targets enzymes that have the greatest control on fluxes.

## Background

A primary goal of molecular evolutionary studies is to elucidate the driving forces governing evolutionary change and mechanisms of molecular evolution. A general pattern arising from previous studies is that genes or proteins varied substantially in their evolutionary rates, spanning more than 3 orders of magnitude [1-4]. Although many determinants, including functional, biophysical, and fitness-related variables [3,5,6], have been proposed to explain such variation, what determines the evolutionary rate of a protein has been debated for decades and remains largely elusive despite some large-scale investigations [4,7,8]. One possible reason for this is that many studies have focused on protein interaction networks that are very heterogeneous with different types of interactions [5,8]. False correlations in protein interaction networks and noise in biological data further complicate the analyses of protein evolution [4,6]. To study evolutionary rates and patterns of proteins in well-characterized metabolic networks or pathways has thus become an alternative for understanding of molecular evolution [9-15].

Analysis of molecular evolutionary patterns of different genes within metabolic pathways allows us to determine whether these genes are subject to equivalent evolutionary forces and how natural selection shapes the evolution of proteins in an interacting system. In studies of evolutionary rates of genes in the plant anthocyanin pathway, Rausher et al. [11] and Lu and Rausher [13] demonstrated that upstream genes in the pathway evolved more slowly than downstream genes both over a broad taxonomic distance involving monocots and dicots and at the intragenic level between species within a genus. They suggested that such difference in evolutionary rates between upstream and downstream genes was due to more constraint upon the upstream genes because they participated in several different biochemical pathways. The hypothesis that earlier acting genes in genetic pathways are under strong purifying selection has been confirmed by some studies (e.g., [9,16]) but not been supported by the others (e.g., [12] and see [17]).

Another hypothesis regarding protein evolution involves a theory of pathway fluxes, indicates that natural selection would target enzymes that control metabolic fluxes and thus where selection operates in a pathway will depend on the distribution of flux control among pathway genes [17,18]. It has been suggested that pathway branch points are usually the targets of selection because they might control the expression of biochemical phenotypes disproportionately [10,14,18]. A third alternative hypothesis argued that translational selection and selection for protein folding and design might govern the rate of protein sequence evolution [7,19-21]. Despite these arguments and some other attempts [4,5,8,22], empirical studies with specific metabolic networks or pathways remain limited, particularly for plants.

**Figure 1:**
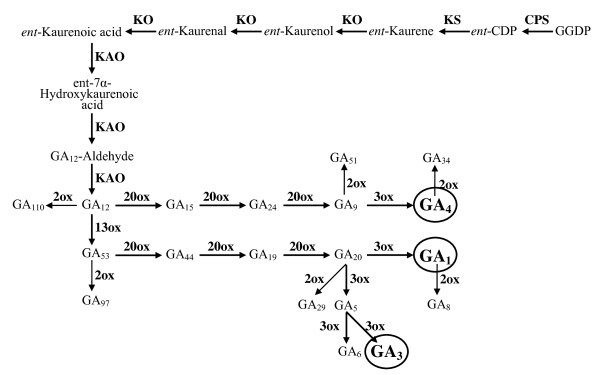
**Simplified GA biosynthetic pathway in plants**. The shaded metabolites have been demonstrated to function as bioactive hormones.

Gibberellin (GA) biosynthetic pathway is involved in the production of gibberellins that control many aspects of plant growth and development, including seed germination, stem elongation, leaf expansion, and flower and seed development [23,24]. Despite a hundred GAs identified from plants, a small number of them such as GA_1 _and GA_4 _are found to function as bioactive hormones. These bioactive GAs are synthesized from trans-geranylgeranyl diphosphate (GGDP) through the 12-step conversion (Figure 1). To date 7 enzymes have been identified to be responsible for GA biosynthesis and function in multiple locations within the cell, involving the chloroplast, the ER membrane, and the cytoplasm. These 7 enzymes can be divided into 2 groups, i.e., the enzymes catalyzing early steps: *ent*-copalyl diphosphate synthase (CPS), *ent*-kaurene synthase (KS), *ent*-kaurene oxidase (KO), and *ent*-kaurenoic acid oxidase (KAO) and those for later steps: GA20-oxidase (GA20ox), GA3-oxidase (GA3ox), and GA2-oxidase (GA2ox) (Figure 1) [23]. In addition to a well-defined pathway, almost all of the genes encoding the 7 metabolic enzymes and their related mutants have been isolated and identified [23,24]. Therefore, the GA biosynthetic pathway provides an excellent system for exploring the correlation between evolutionary rate and position of the genes in metabolic pathways as well as its implications in molecular evolution. In addition, the GA biosynthetic pathway is also a good system to investigate the relative importance of selective constraint and positive selection as well as how adaptive variation is distributed among proteins in metabolic pathways. In this study, we isolated and sequenced portions of the 7 GA pathway genes from representative species of the rice tribe and analyzed their rates and patterns of molecular evolution. Our specific goals were 1) to compare evolutionary rates across the GA pathway genes and ask if evolutionary rate of the genes depends on its function or gene's position within the pathway; 2) to examine whether the GA pathway genes exhibit evidence of positive selection and particularly to understand the relative contribution of selective constraint and positive selection to variation of evolutionary rates of the GA pathway genes.

## Methods

### Plant materials and genes analyzed

We isolated and sequenced portions of all 7 GA pathway genes from members of the rice tribe (Oryzeae). Phylogeny of the rice tribe has been well resolved by previous studies based on sequences of plastid, mitochondrial and nuclear genes [25,26] and provided an important framework for molecular evolution study for this group (Additional file 1). To analyze the patterns of molecular evolution of the GA pathway genes, 8 diploid species were selected to represent major phylogenetic lineages of Oryzeae that are separated by a range of genetic distances [26]. They consisted of 5 *Oryza *species representing 5 diploid genome types, *Oryza sativa *(A), *O. officinalis *(C), *O. australiensis *(E), *O. brachyantha *(F), *O. granulata *(G), and one each of other 3 genera in the tribe (*Chikusichloa aquatica*, *Luziola leiocarpa*, and *Rhynchoryza subulata*). One species in the tribe Ehrhartoideae that is sister to Oryzeae, *Ehrharta erecta*, was used as an outgroup [26,27].

Of 7 enzymes in the GA biosynthetic pathway, 4 enzymes in the early steps (CPS, KS, KO and KAO) were encoded by single-copy genes (*CPS1*, *KS1*, *KO2 *and *KAO*) [23] and sampled in this study. Because the 3 enzymes in the later steps (GA20ox, GA3ox and GA2ox) were encoded by small gene families [23,24], we chose to sequence *GA20ox2*, *GA3ox2 *and *GA2ox4 *that were highly expressed in all organs of the wild-type rice [23]. Previous studies showed that mutants with all 7 enzymes except for GA2ox exhibited a typical phenotype of dwarfism, indicating the functional importance of these enzymes in GA biosynthesis [23]. Detailed information of the genes and the sequences and positions of the primers are listed in table 1 and Additional file 2, respectively. Species sampled and the GenBank accession numbers of the sequences obtained in this study are listed in Additional files 3 and 4, respectively.

**Table 1:** Information on the genes and sequences of the primers used in the study.

Gene	Location^*a*^	Enzyme	Aligned coding length (bp)	GC (GC3)^*b*^	ENC^*b*^	Primer sequences^*c*^
*CPS1*	2	*ent*-copalyl diphosphate synthases I	1002	0.462 (0.494)	54.6	F1: AACTTGTGGAGGTTAGCAG F2: TGTGGAGGYTAGCRGAGG R1: AAGTCGCTCAGAGGCACG R2: TAGCCCATGCAAGTCGCTC
*KS1*	4	*ent*-kaurene synthase I	1023	0.426 (0.417)	52.8	F1: TGCTGAAGCTTCCAGTTTCC F2: TCCAGTTTCCGTGAATCAC R1: CTTGCACATCTTCCAGAAC R2: CCTTGACGACTGCATTCAC
*KO2*	6	*ent*-kaurene oxidase II	1050	0.514 (0.639)	58.2	F: CTGTAGTTGTGCTCAATTC R1: GCCATCGTCTTGTACATGTC R2: TCAGCCTCCACYCGAACTC
*KAO*	6	*ent*-kaurenoic acid oxidase	1053	0.603 (0.820)	39.2	F: CAGGACGTTCATGTTCAGCAG R1: TCGTCGCCAAGCAGTTGTC R2: GCCAAGCAGTTGTCCAC
*GA20ox2*	1	GA20-oxidase II	597	0.714 (0.966)	30.1	F: ATCCCGGAGCCATTCGTSTG R: TGAAGGTGTCGCCGATGTTG
*GA3ox2*	1	GA3-oxidase II	786	0.710 (0.965)	30.4	F1: ACCCGCTCTRCTTCGACTTC F2: GGCGGGTGCCGGAGACGCACG R: CCATGTACTCSGGCCACGTGAC
*GA2ox4*	5	GA2-oxidase IV	819	0.694 (0.926)	34.0	F: GAGCAGATCTCGCTGSTGAG R1: CAGGCGGTTGTCGCYGAG R2: AGGCGGGAGAGGTAGGCAG
Total			6330			

^*a*^Chromosomal locations in *O. sativa*.

^*b*^Average of 8 Oryzeae species.

^*c*^Locations of the primers for each fragment are showed in Additional file 2. The internal primers were used for cycle sequencing are listed in Additional file 5.

### DNA extraction, amplification and sequencing

Total DNA was isolated from silica-gel dried or fresh leaves using the method as described by Ge et al. [25]. Because the DNA fragments of each gene contained multiple exons and introns, additional internal primers were used for sequencing of four genes (*CPS1*, *KS1*, *KO2 *and *KAO*) that were about 2 kb in length. Their sequences and melting temperatures (Tm) are provided in Additional file 5. Polymerase chain reaction (PCR) was performed in a total volume of 25 μl which contained 5–50 ng of template DNA, 0.2 μM of each primer, 200 μM of each dNTP, 10 mM Tris-Cl (pH 8.3), 50 mM KCl, 1.5 mM MgCl_2_, and 0.75 U ExTaq DNA polymerase (TaKaRa, Shiga, Japan). Amplification was carried out in a T gradient 96 U thermocycler (Biometre, Göttingen, Germany) as follows: 3 min at 94°C, followed by 33 cycles of 30 ~ 35 s at 94°C, 30 ~ 35 s at 54°C ~ 58°C, 2 min 30 s at 72°C and a final extension at 72°C for 10 min (*CPS1*, *KS1*, *KO2*, and *KAO*). Because GC contents of *GA20ox2*, *GA3ox2 *and *GA2ox4 *were as high as about 70%, routine PCR amplification did not work well. We performed the amplification using LA Taq DNA polymerase, special GC Buffer I and dNTP Mixture (TaKaRa, Shiga, Japan). Except for annealing temperature at 60°C, the protocol was basically similar to the above protocol. All PCR products were separated by electrophoresis on 1.5% agarose gels stained with ethidium bromide. After purification using DNA Purification kit, most of amplification products were sequenced directly. For those PCR products that were weakly amplified and difficult to be sequenced directly, we used the pGEM-T Vector (Promega Corporation, Madison, USA) to clone and sequence at least three clones. Sequencing was carried out on a Megabase 1000 automatic DNA sequencer (Amersham Pharmacia Biotech). All sequences have been deposited in GenBank, and their accession numbers are EF577637 ~ EF577669, EU179376 ~ EU179435 (Additional file 4).

### Sequence analyses

Sequences were translated into the predicted amino acid sequence and aligned using ClustalX with version 1.81 [28], followed by manual adjustment. Excluding intron regions, the stretches of amino acid residues were conserved well enough across eight species to perform unambiguous alignments. The possibility of sequence saturation was examined using DAMBE 4.5.45 [29]. A plot of the number of transitions and transversions vs. divergence offers a visual display of substitution saturation, with an asymptotic relationship indicating the presence of saturation [29]. Pairwise number of synonymous and nonsynonymous substitutions per site (*d*_S _and *d*_N_) as well as nonsynonymous/synonymous substitution rate ratio (*d*_N_/*d*_S_) between the eight species were calculated for the coding regions of all 7 genes using the improved approximate method of Yang and Nielsen [30], implemented in YN00 (CODEML) of PAML version 4 [31]. This method considers two important evolutionary features of DNA sequence: transition/transversion rate bias and codon frequency bias. Based on the pruned phylogenetic tree of the eight species (Additional file 6), we also estimated the *d*_N_/*d*_S _value of each gene using CODEML of the PAML version 4 [31].

The magnitude of codon bias is often used to reflect the degree of selective constraint in a gene and variation of synonymous substitution rates among genes may be related to codon usage [32,33]. To measure the extent of codon usage bias, we estimated effective number of codons (ENC) using DnaSP version 4.10.9 [34], which varies between 20 and 61, with the lower the value, the more biased codon usage [35].

### Comparing *d*_S _and *d*_N _among genes

The pairwise synonymous and nonsynonymous genetic distances of 8 species were calculated for each gene using the method of Yang and Nielsen [30]. Of total 28 species pairs, we chose 15 species pairs involving 5 *Oryza *species and other three representatives of the rice tribe to represent maximum divergence time (20 myr, Additional file 1). To evaluate the reliability of estimated genetic distances of the 15 pairs, we checked the distance variance of each pair of the genes and found that the pairs involving *O. sativa *or *O. brachyantha *have high variance for *GA20ox*, *GA3ox *and *GA2ox*. Thus six species pairs involving either of the two species were excluded. Based on the rest 9 species pairs (*O. officinalis vs. C. aquatica*, *O. officinalis vs. L. leiocarpa*, *O. officinalis vs. R. subulata*, *O. australiensis vs. C. aquatica*, *O. australiensis vs. L. leiocarpa*, *O. australiensis vs. R. subulata*, *O. granulata vs. C. aquatica*, *O. granulata vs. L. leiocarpa*, and *O. granulata vs. R. subulata*), the mean distance and its standard error of each gene were calculated to show variation of *d*_S_, *d*_N _among genes. To compare *d*_S _and *d*_N _among genes, we calculated the *d*_S _and *d*_N _for each branch of the pruned tree of the six species (Additional file 1 and Additional file 6) using CODEML of the PAML version 4 [31]. Of the tree, there are total 10 evolutionary independent branches (Additional file 6). Two data matrices for seven genes were obtained for *d*_S _and *d*_N_, respectively. We used two approaches to detect whether evolutionary rates differed among the genes. First, we used multiple range test and Fisher's least significant difference (LSD) procedure to discriminate the means of the genetic distances of 9 species pairs among 7 genes. To compare *d*_S _and *d*_N _among genes, we calculated the *d*_S _and *d*_N _for each branch of the pruned tree including six species (Additional file 6) using CODEML of the PAML version 4 [31]. On the tree, there are 10 evolutionarily independent branches (Additional file 6). Then we used Wilcoxon signed rank test [36] in the pairwise comparisons of distances of the 10 branches between genes to test significant difference among the GA genes.

### Comparing *d*_N_/*d*_S _among genes

Pairwise *d*_N_/*d*_S _ratios of 8 species were estimated for each gene using the method of Yang and Nielsen [30]. The mean *d*_N_/*d*_S _ratios of 9 species pairs and standard errors were calculated to show variation of *d*_N_/*d*_S _among genes. We used a method described by Lu and Rausher [13] to compare *d*_N_/*d*_S_ratios for the seven genes. For this analysis, we first estimated a single value of *d*_N_/*d*_S _for each gene using the model M0 of by CODEML of PAML version 4 [31]. We then detected the significance of differences of *d*_N_/*d*_S _values between genes by comparing the likelihood of the model using the estimated value of *d*_N_/*d*_S _to the likelihoods of the same model and using *d*_N_/*d*_S _constrained to various values [13].

### Detection of positive selection

The ratio of nonsynonymous to synonymous substitution rate (ω = *d*_N_/*d*_S_) provides an effective measure to detect selection or selective pressure on a gene or gene region, with ω < 1, = 1, and > 1 indicating purifying selection, neutral evolution, and positive selection, respectively [31,37]. We performed likelihood-based analyses using the CODEML program of PAML version 4 [31] to explore the selective processes acting on the GA biosynthetic genes. First, we tested whether the evolutionary rates of each gene in the pathway differed among lineages within the gene tree by using the branch models. The one ratio model (M0) assumes a single ω for all branches and all sites, whereas the free ratio model (Mf) assumes an independent ω ratio for each branch of the tree. A likelihood ratio test (LRT) was conducted to determine whether there was statistically significant difference between two models. If the LRT is significant, the null hypothesis that two models are not significantly different is rejected, and the model with higher likelihood value is assumed to be a better model [38,39].

We next used site-specific models to examine whether particular amino acid residues were subject to positive selection because the branch test often has little power to detect positive selection due to the fact that the ω ratio is seldom detected greater than 1 if all the sites are averaged [40]. The nested codon models [39,40] were performed for each gene separately. In addition to one ratio model (M0), the neutral model (M1a) classifies all the sites into 2 categories, one under strict constraint (0 < ω < 1) (purifying selection) and the other under neutral (ω = 1). Positive selection model (M2a) is based on M1a and assumes a third category under positive selection (ω > 1). Beta model (M7) assumes a beta distribution of the ω ratios, and beta&ω model (M8) extends an independent ratio estimated by the data. Models assuming positive selection M8 and M2a are compared with null models M7 and M1a, respectively. Positive selection is invoked if the LRT is significant and there is site with ω > 1 [39,40].

## Results

Sequences of the 7 GA pathway genes were isolated from all sampled Oryzeae species and an outgroup, *Ehrharta erecta*. The sequenced regions of the 7 genes ranged from 699 bp to 2231 bp in rice and their aligned coding regions varied between 597 bp and 1053 bp for species used in this study (table 1 and Additional file 4). The GC contents for the total and 3 individual codon positions were similar for the same gene across species but varied greatly among genes, with the means ranging from 0.426 (*KS1*) to 0.714 (*GA20ox2*) (table 1 and Additional file 4). It is noted that GC contents of the 3 genes at the later steps were much higher than those of the 4 genes at the early steps in the pathway, particularly at the 3rd position (GC3) where the averages were 0.926 ~ 0.966 for the genes at the later steps in contrast to the values of 0.417 ~ 0.82 for the genes at the early steps (table 1 and Additional file 4). To estimate accurately and compare substitution rate and ω ratios of different GA pathway genes, we evaluated sequence saturation of substitution for each of the seven genes. Results of saturation plots did not reveal any mutational saturation for all the seven GA genes, indicating that the datasets were suitable for further analyses of the differences of substitution rate and ω ratios.

### Comparing *d*_N _and *d*_S _among genes

**Figure 2:**
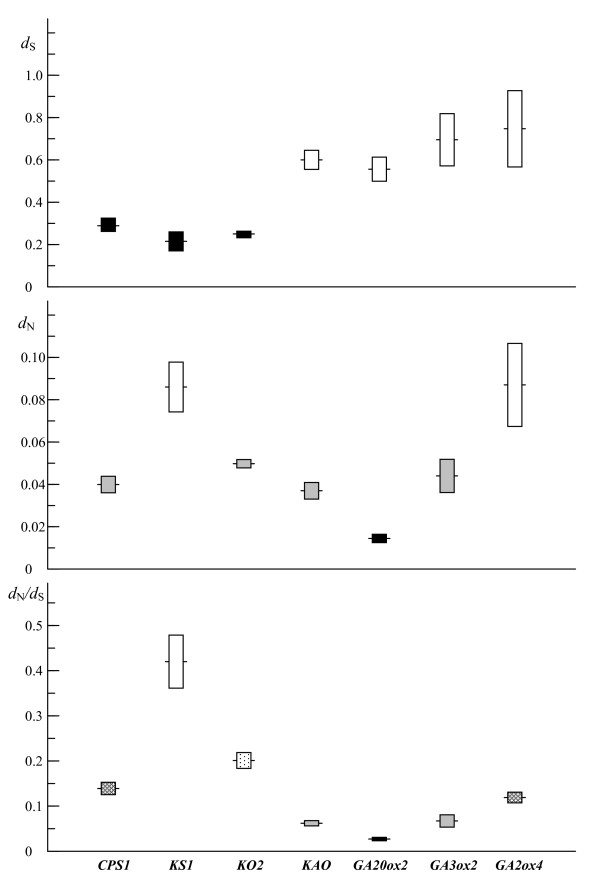
**Evolutionary rates at sysnonymous and nonsynonymous sites of the GA pathway genes**. Horizontal bars indicate the averages of *d*_S_, *d*_N _and *d*_S_/*d*_N _values. Boxes represent 95% confidence intervals.

The averages of *d*_S _and *d*_N _of the 9 species pairs for each gene were shown in Figure 2 and table 2. Both *d*_S _and *d*_N _values varied greatly among genes, with the highest *d*_S _(0.747 for *GA2ox4*) being 3.5 times the lowest (0.214 for *KS1*) and the highest *d*_N _(0.086 for *KS1*) being 5.9 times the lowest (0.015 for *GA20ox2*). For *d*_S_, the 3 genes in the early steps of the pathway (*CPS1*, *KS1 *and *KO2*) have significantly lower *d*_S _values than *KAO *and 3 genes at the later steps of the pathway (*GA20ox2*, *GA3ox2 *and *GA2ox4*). For *d*_N_, *GA20ox2 *has the lowest value (0.015), followed by 4 genes (*KO2*, *GA3ox2*, *CPS1*, and *KAO*), and the second and last genes in the pathway, *KS1 *and *GA2ox4*, have the highest and comparable *d*_N _value (0.086 and 0.087). Unlike *d*_S _values, *d*_N _varied significantly both between and within genes in the early and late steps of the pathway. It is noted that 2 types of substitutions did not correlated (r^2 ^= 0.009, *P *= 0.840), which is clearly reflected by the fact that *KS1 *had a highest *d*_N _value but the lowest *d*_S _(Figure 2 and table 2). Multiple range tests generated 2 and 3 homogenous groups of genes for *d*_S _and *d*_N_, respectively, with statistically significant difference between genes among groups and non-significant difference between genes within groups (table 2). To overcome the rate heterogeneity of *d*_S _and *d*_N _among lineages, we performed the Wilcoxon signed rank test for pairwise comparisons of *d*_S _and *d*_N _between genes for 10 independent branch of pruned tree consisting of 6 species. For *d*_S_, the gene *KAO *and three late-step genes (*GA20ox2*, *GA3ox2*, *GA2ox4*) showed significant rate differences from the first 3 genes in the pathway (*CPS1*, *KS1 *and *KO2*) (*P *< 0.01). No significant difference was detected between *KAO *and the late-step genes and among the early-step genes. For *d*_N_, none of the tests showed significant difference, involving the comparisons between and within both early- and late-step genes (table 2 and Additional file 7).

**Table 2:** Tests on difference of *d*_S_, *d*_N _and *d*_N_/*d*_S _among the 7 GA genes^a^

Gene	*d*_S_		*d*_N_		*d*_N_/*d*_S_	
	Mean ± SE^b^	WSRT^c^	Mean ± SE^b^	WSRT^c^	Mean ± SE	ω^d^
*CPS1*	0.292 ± 0.016	A	0.040 ± 0.002	B	0.139 ± 0.007	C (0.144)
*KS1*	0.214 ± 0.023	A	0.086 ± 0.006	C	0.420 ± 0.030	D (0.386)
*KO2*	0.250 ± 0.008	A	0.050 ± 0.001	B	0.201 ± 0.009	C (0.177)
*KAO*	0.600 ± 0.023	B	0.037 ± 0.002	B	0.062 ± 0.003	B (0.074)
*GA20ox2*	0.556 ± 0.029	B	0.015 ± 0.001	A	0.027 ± 0.002	A (0.022)
*GA3ox2*	0.699 ± 0.063	B	0.044 ± 0.004	B	0.066 ± 0.007	B (0.073)
*GA2ox4*	0.747 ± 0.092	B	0.087 ± 0.010	C	0.119 ± 0.006	C (0.129)

^a ^Capital letters (A to D) indicate the homogenous groups.

^b ^Calculated based on 9 specie pairs.

^c ^Wilcoxon signed rank test using 10 independent branches of the pruned tree consisting of 6 species

^d ^ω, calculated as *d*_N_/*d*_S _ratios using PAML model M0.

### Comparing *d*_N_/*d*_S _among genes

The *d*_N_/*d*_S _ratios of the 9 species pairs varied remarkably among the 7 genes, with the highest value (0.420 for *KS1*) being 15.6 times the lowest (0.027 for *GA20ox2*) (Figure 2 and table 2). We compared *d*_N_/*d*_S _ratios for the 7 genes and detected the significance of differences between genes using the method of Lu and Rausher [13]. The *d*_N_/*d*_S _value for each gene ranged from 0.386 (*KS1*) to 0.022 (*GA20ox2*) with a trend similar to that estimated by pairwise *d*_N_/*d*_S _ratios (table 2). Based on log-likelihood ratio comparisons, we found 4 homogenous groups, i.e., A (*GA20ox2*), B (*KAO *and *GA3ox2*), C (*KO2*, *CPS1*, and *GA2ox4*), and D (*KS1*). As shown in table 3, significant difference of *d*_N_/*d*_S _ratios is found between genes from different groups but not found between genes from the same groups. The results are similar to those obtained by multiple range test and Wilcoxon signed rank test, with the exception that *KO2 *and *CPS1 *were in the same group in log-likelihood ratio comparisons (table 3).

**Table 3:** Comparison and significant test of log-likelihood ratio of *d*_N_/*d*_S _between genes

Category/Gene	Estimated *d*_N_/*d*_S_	lnL	Fixed *d*_N_/*d*_S_	lnL	2ΔL^a^
Between groups D and C					

*KS1*	0.386	-3341.16	0.2815	-3345.46	8.59**
*KO2*	0.177	-3118.44	0.2815	-3126.22	15.56***
*CPS1*	0.144	-3034.22	0.2815	-3049.87	31.3***
*GA2ox4*	0.129	-2733.05	0.2815	-2755.45	44.8***
Within group C					
*KO2*	0.177	-3118.44	0.153	-3119.19	1.50^NS^
*CPS1*	0.144	-3034.22	0.153	-3034.35	0.26^NS^
*GA2ox4*	0.129	-2733.05	0.153	-2734.14	2.18^NS^
Between groups C and B					
*KO2*	0.177	-3118.44	0.1015	-3128.93	20.98***
*CPS1*	0.144	-3034.22	0.1015	-3038.05	7.66**
*GA2ox4*	0.129	-2733.05	0.1015	-2735.16	4.21*
*KAO*	0.074	-3071.99	0.1015	-3075.8	7.62**
*GA3ox2*	0.073	-2210.46	0.1015	-2213.21	5.50*
Within group B					
*KAO*	0.074	-3071.99	0.0735	-3071.99	0^NS^
*GA3ox2*	0.073	-2210.46	0.0735	-2210.46	0^NS^
Between groups B and A					
*KAO*	0.074	-3071.99	0.0475	-3078.8	13.62***
*GA3ox2*	0.073	-2210.46	0.0475	-2214.72	8.52**
*GA20ox2*	0.022	-1373.16	0.0475	-1379.06	11.8***

^a ^d.f. = 1, ***P < 0.001, ** *P *< 0.01, *P < 0.05, ^NS ^not significant.

### Codon usage bias and its correlation with GC3 and substitution rates

Estimates of the codon usage indicated that the ENC for the same gene was similar across all species (χ^2 ^test, *P *= 1.00) (Additional file 4), suggesting that the magnitude of codon bias underwent little change during the diversification of the rice tribe. However, substantial variation was found among genes in the degree of codon bias, with the means of ENC ranging from 30.1 (*GA20ox2*) to 58.2 (*KO2*) (table 1 and Additional file 4). Pairwise comparisons of one-way ANOVA found that differences of the ENC were significant for all pairwise comparisons (*P *< 0.001) except for *CPS1 *vs. *KS1 *(*P *= 0.090) and *GA20ox2 *vs. *GA3ox2 *(*P *= 0.386). Note that the 3 genes in the late steps (*GA20ox2*, *GA3ox2*, and *GA2ox4*) exhibited strong codon usage bias (ENC = 30.1 ~ 34.0), whereas the 3 genes in the early steps (*CPS1*, *KS1 *and *KO2*) possessed little codon usage bias (ENC = 52.8 ~ 58.2). *KAO *exhibits an intermediate degree of codon usage bias with ENC of 39.2. ANOVA showed that ENC of the late-step genes differed significantly from those of the early step genes (*P *< 0.001). To further investigate the correlation between evolutionary rate and expression level, we analyzed the correlation between each of three parameters (*d*_N_, *d*_S_, and *d*_N_/*d*_S_) and ENC that negatively correlates with expression level. Results showed that significantly negative correlation existed between *d*_S _and ENC (*P *< 0.001), but not between ENC and either *d*_N _(*P *= 0.540) or *d*_N_/*d*_S _(*P *= 0.103).

### Testing for positive selection

Significant rate heterogeneity among the GA pathway genes, particularly for *d*_N_/*d*_S _ratios that span more than one order of magnitude (Figure 2), might result either from intense purifying selection on slowly evolving genes (e.g., *GA20ox2*) or from frequent episodes of positive selection on fast evolving genes (e.g., *KS1*). Therefore, we first used branch models to test whether the evolutionary rate of each gene differed among lineages within the rice tribe. For all 7 genes, free ratio model (Mf) did no have significantly higher likelihood scores than one ratio model (M0) (*P *> 0.05) (Additional file 8). The ω values were estimated to be 0.022 ~ 0.386 under M0 model, suggesting that purifying selection or selection constraint best explains the evolution of these genes.

Because the branch model test averages the ratio across all sites and is a very conservative test of positive selection [39,40], we applied site-specific codon models to the 7 genes separately, to determine whether there was positive selection on codon sites of these genes. Results showed that, for all 7 genes, models M2a and M8 assuming positive selection were not significantly better than the null models M1a and M7 (for M1a vs. M2a, 2ΔL = 0.0 ~ 3.72, 0.1 <*P *≤ 1.0; for M7 vs. M8, 2ΔL = 0.0 ~ 5.3, 0.05 <*P *≤ 1.0) (Additional file 8). Consequently, all 7 genes are under strong selective constraint, which excludes the possibility of past episodes of positive selection on these genes.

## Discussion

### Evolutionary rate and position of genes in the pathway

We used two different approaches to have detected significant rate heterogeneity among 7 GA pathway genes for both synonymous and nonsynonymous sites. Multiple range tests classified the 7 genes into 2 and 3 homogenous groups of synonymous and nonsynonymous sites, respectively, with significant rate difference among genes between groups but not among genes within groups. It is noteworthy that the patterns of rate heterogeneity were different for 2 types of substitutions. For *d*_S_, the late-step genes (*GA20ox2*, *GA3ox2*, and *GA2ox4*) have significantly higher substitution rates than the early-step genes (*CPS1*, *KS1*, *KO2*, and *KAO*) (*P *< 0.05), whereas one early-step gene (*KS1*) and one late-step gene (*GA2ox4*) consist of one homogenous group with the highest *d*_N_.

It is well established that large variation in among-gene substitution rates might be determined by two factors: the rate of mutation and the intensity of selection [1,5,41]. To distinguish these two possibilities, we first asked whether there was regional similarity for *d*_S _and *d*_N _values because mutation pressure would lead to region-specific mutation rates[1,42]. It is evident that genes in the same homogenous *d*_S _or *d*_N _groups locate on different chromosomes (table 1). These observations, in conjunction with the lack of correlation between *d*_S _and *d*_N_, do not support the mutation-driven hypothesis. To further investigate possible role of mutation, we calculated the GC content of noncoding sites of the 7 genes (Additional file 4). If difference of GC3 was caused by mutation pressure, we would expect a positive correlation between GC3 of coding sites and GC of noncoding sites because the latter is expected to reflect differences in mutation rate [2,5]. The result did not find a significant correlation between them (r^2 ^= 0.516, *P *= 0.069), suggesting that mutation bias alone cannot explain the differences of codon usage in the 7 genes. Consequently, differences of selection intensity are most likely to account for the substitution rate variation in the GA pathway genes, though differential mutation pressures on genes cannot be excluded entirely.

Rausher et al. [11] studied evolution patterns of 6 core structural genes in the anthocyanin pathway and found that, over a broad taxonomic distance involving monocots and dicots, the upstream genes evolved substantially more slowly than the downstream genes. They thus hypothesized that the upstream genes in a metabolic pathway evolved more slowly than the downstream genes due to stronger purifying selection for the upstream genes. Such a pattern has also been confirmed at the intragenic [13] and population levels [15] in the genus *Ipomoea *as well as by a molecular population study on regulatory and signal transduction genes [9] but was not supported by other studies [8,12,17]. In the GA metabolic pathway, we did not find the correlation between substitution rate and position of genes. Notably, 2 genes (*KS1 *and *GA20x4*) with the highest *d*_N _belong to the early and later acting genes, respectively (Figure 1). In addition, 3 late-step genes have significantly lower *d*_N_/*d*_S _ratios than the early-step genes, contrary to the pattern found in anthocyanin pathway genes [13]. Particularly, the ENC data indicated that silent sites were more constrained (lower ENC values) in the downstream than in the upstream genes (table 1), which would overestimate *d*_N_/*d*_S _ratios of the downstream genes. This observation suggests that the difference of selective constraints between the downstream and upstream genes would be much more striking.

As indicated by Cork and Purugganan [17], the specific nature of selection on the component genes depended largely on the function of the pathway but the dichotomy between upstream and downstream genes in a pathway was a crude differentiation of function. In our case, no correlation between evolutionary rate and position of genes in the GA pathway seems better explained by the pathway fluxes hypothesis, suggesting that natural selection more likely target enzymes that control metabolic fluxes [17,18]. As predicted by this theory, the enzymes carrying high metabolic fluxes (e.g., at branch points of the pathway) will control disproportionately the expression of biochemical phenotypes and experience higher evolutionary constraints [8,14,17]. In the GA pathway, enzymes that catalyze multiple steps or at the branching points (KO, KAO, GA20ox2, GA3ox, and GA2ox) have also low *d*_N_/*d*_S _values (Figures 1 and 2). In contrast, the gene with the highest *d*_N_/*d*_S _encodes the enzyme involving in a single step (KS) in the pathway. It should be noted that upstream enzymes in many pathways are positioned above major branch points and thus suffer from stronger selection constraints due to pleiotropic effects [5,17]. This may partly explain, in terms of pathway fluxes theory, the differences in levels of selective constraints upon upstream and downstream genes observed in earlier studies [9,11,13,15]. In addition, we found that the genes at the branching points (e.g., *GA20ox2 *and *GA3ox2*) have high levels of gene expression (usually measured by codon usage bias) (table 1) and slow *d*_N _(Figure 2). Such a correlation in the GA pathway also supports the functional hypothesis that more highly expressed protein will consume a larger proportion of the cell resources and might control more important metabolic fluxes [6]. This explanation is in well agreement with studies on the GA pathway genes in other plants. For example, increased expression of a *GA20ox *gene in transgenic *Arabidopsis *plants caused an increase in GA levels and GA overdose phenotypes while overexpression of the *CPS *in *Arabidopsis *(*AtCPS*) did not affect the levels of bioactive GAs or the phenotype [24,40]. It should be noted that translation selection and selection for protein folding might be also a factor contributing to differences of evolutionary rates [7,21]. However, our case cannot provide additional evidence because of limited number of genes used and lack of information on the protein structure and expression of these genes.

### Rate variation among GA genes is attributed to selective constraint rather than positive selection

As discussed above, we excluded the possibility that difference of mutation rates was the main factor for rate variation of the GA pathway genes. However, high *d*_N_/*d*_S _ratio for some genes (e.g., *KS1*, *KO2*) might be due to relaxation of selection and could also be explained by repeated adaptive selection on these proteins. Earlier studies have showed that evolutionary rate variation among proteins might be attributed either to the differences in the magnitude of selective constraints [1,11,15,41] or to the differences in the frequency of positive selection [37]. In spite of many investigations on factors influencing rate of protein evolution [5,6,8,14,17], few have been conducted to explore the relative contribution of differential selective constraint and differential positive selection to rate variation of pathway genes [13,15]. In this study, both branch and codon models failed to detect any signature of positive selection for any lineage and codon of the GA pathway genes, suggesting that increased rate of nonsynonymous substitution in some genes is mainly ascribed to relaxed selective constraint, in agreement with the studies on anthocyanin pathway genes [13,15]. Unlike some empirical studies [14], however, we did not detect adaptive substitution for those genes that encode enzymes controlling metabolic fluxes.

Studies showed that highly expressed genes were more important to an organism and thus subject to greater selective constraint and these genes usually had greater codon bias because of selection for translation efficiency [5,13,33,43,44]. A recent study on a set of six distantly related model organisms confirmed that translational selection would be an important mechanism behind the constraints of proteins by increasing translational accuracy and translational robustness [21]. Therefore, the degree to which a gene is subject to selective constraint could also be reflected by the magnitude of codon usage bias in that gene. Indeed, *GA20ox2 *that is identical to the rice Green Revolution gene, *Semi-Dwarf1 *[23], has the lowest nonsynonymous substitution rate (the smallest *d*_N_/*d*_S _ratio) among 7 genes surveyed. This gene exhibits the strongest codon usage bias (the lowest ENC), consistent with its functional importance. In contrast, the gene (*KS1*) that encodes the enzyme involving a single step in the pathway has the highest *d*_N_/*d*_S _ratio and much weaker codon usage bias (much higher ENC). Consequently, the significant heterogeneity of evolutionary rate of the GA pathway genes is mainly ascribed to differential constraint relaxation rather than the positive selection. It should be noted, however, that the power to detect positive selection using the above methods may be low, particularly when adaptive substitutions are distributed across many amino acid sites such that *d*_N_/*d*_S _is not elevated above 1 for any site [15,45]. Further investigations with alternative tests on within-species variation [10,12,14,15] would be necessary to detect evidence of positive selection and elucidate the differential rate of adaptive substitution among the GA pathway genes.

## Conclusion

Based on sequence analyses of 7 core structural genes of the gibberellin biosynthetic pathway, we have detected significant rate heterogeneity among these genes for both synonymous and nonsynonymous sites and ascribed such rate variation to differences of selection intensity rather than differential mutation pressures on the genes. Our study demonstrates that the downstream genes in the GA pathway did not exhibit the elevated substitution rate, inconsistent with previous argument that downstream genes in metabolic pathways would evolve more rapidly than upstream genes. Instead, we found that the GA pathway genes encoding either the enzyme at the branch point or enzymes catalyzing multiple steps had the lowest evolutionary rates because of strong purifying selection, supporting the pathway flux theory that predicts that natural selection primarily targets enzymes that have the greatest control on fluxes.

## Abbreviations

GA: gibberellin; ENC: effective number of codons; LRT: likelihood ratio test.

## Authors' contributions

SG and YHY designed the research and outlined the manuscript together. YHY and SG performed the research. YHY and FMZ analyzed the data. SG, YHY and ZFM interpreted the data and wrote the paper. All authors have read and approved the final manuscript.

## Supplementary Material

Additional file 1 **figure S1**. Phylogeny of the rice tribe (Oryzeae) obtained from the combined *Adh2 *and *GPA1 *sequences by Bayesian inference under TrN+G model [26]. Bold faces indicate the species sampled in this study. Click here for file

Additional file 2 **figure S2**. Schematic diagrams of 7 GA biosynthetic genes and the regions sequenced in this study. Boxes and lines indicate exons and introns, respectively. Exon numbers are labeled with the roman numbers. Locations of primers for each fragment are showed above the diagrams and their sequences are provided in table 1. Click here for file

Additional file 3 **table S1**. Species used in this study. Click here for file

Additional file 4 **table S2**. Information of the 7 GA genes sampled in the present study. Click here for file

Additional file 5 **table S3**. Sequences and melting temperatures (Tm) of internal primers used for sequencing of four genes that are ~2 kb in length. Click here for file

Additional file 6 **figure S3**. Pruned phylogeny tree consisting of 6 species. Branches b1 to b10 are 10 independent branches. Click here for file

Additional file 7 **table S4**. Wilcoxon signed rank test of pairwise comparisons of *d*_N _and *d*_S _between genes for 10 independent branches of the pruned tree consisting of 6 species. Click here for file

Additional file 8 **table S5**. Log-likelihood values, ω ratios and parameter estimates under models of variable ω ratios among codon sites. Click here for file
