# Supplementary material for: Evolutionary rate patterns of the Gibberellin pathway genes
Source: BMC Evol Biol. 2009 Aug 18;9:206. doi: 10.1186/1471-2148-9-206 (PMC2794029; doi:10.1186/1471-2148-9-206)
Supplement: Additional file 1 — figure S1. Phylogeny of the rice tribe (Oryzeae) obtained from the combined Adh2 and GPA1 sequences by Bayesian inference under TrN+G model [26]. Bold faces indicate the species sampled in this study. [file 1471-2148-9-206-S1.doc]

### O. sativa

###### O. glaberrima

###### O. meridionalis

###### O. punctata

O. officinalis

###### O. rhizomatis

##### **O. australiensis**

###### Porteresia coarctata

###### Porteresia coarctata

###### O. brachyantha

***O. granulata***

###### Leersia oryzoides

###### Leersia perrieri

###### Leersia hexandra

###### Leersia perrieri

###### Leersia hexandra

###### Leersia oryzoides

**~20 myr**

###### Leersia tisserantti

###### Prosphytochloa prehensilis

###### Potamophila parviflora

## **Chikusichloa aquatica**

###### Rhynchoryza subulata

###### Zizania aquatica

###### Zizania latifolia

###### Zizaniopsis villanensis

***Luziola leiocarpa***

###### Luziola fluitans

###### Ehrharta erecta

10 changes

###### Phyllostachys aurea

**Figure 1**. Phylogeny of the rice tribe (Oryzeae) obtained from the combined *Adh2* and *GPA1* sequences by Bayesian inference under TrN+G model [26]. Bold faces indicate the species sampled in this study.
