# Supplementary material for: Evolutionary rate patterns of the Gibberellin pathway genes
Source: BMC Evol Biol. 2009 Aug 18;9:206. doi: 10.1186/1471-2148-9-206 (PMC2794029; doi:10.1186/1471-2148-9-206)
Supplement: Additional file 2 — figure S2. Schematic diagrams of 7 GA biosynthetic genes and the regions sequenced in this study. Boxes and lines indicate exons and introns, respectively. Exon numbers are labeled with the roman numbers. Locations of primers for each fragment are showed above the diagrams and their sequences are provided in table 1. [file 1471-2148-9-206-S2.doc]

*CPS1*


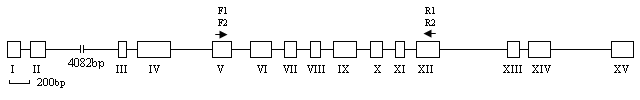


*KS1*


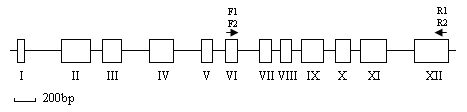


*KO2*


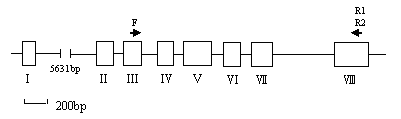


*KAO*


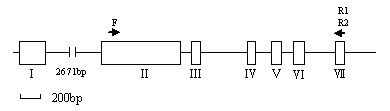


*GA20ox2*


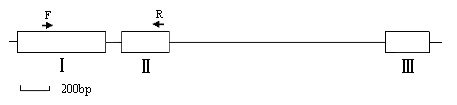


*GA3ox2*

*
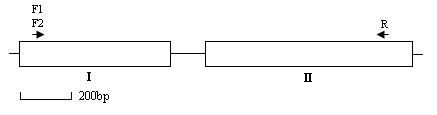
*

*GA2ox4*


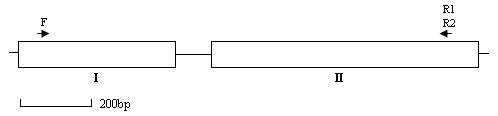


Figure 2 Schematic diagrams of 7 GA biosynthetic genes and the regions sequenced in this study. Boxes and lines indicate exons and introns, respectively. Exon numbers are labeled with the roman numbers. Locations of primers for each fragment are showed above the diagrams and their sequences are provided in table 1.
