# Supplementary material for: Evolutionary rate patterns of the Gibberellin pathway genes
Source: BMC Evol Biol. 2009 Aug 18;9:206. doi: 10.1186/1471-2148-9-206 (PMC2794029; doi:10.1186/1471-2148-9-206)
Supplement: Additional file 3 — table S1. Species used in this study. [file 1471-2148-9-206-S3.doc]

Table 1. Species used in this study.

| Species | Genome | Accession number ^a^ | Origin |
| --- | --- | --- | --- |
| *Oryza sativa* | A | Nipponbare | Japan |
| *O. officinalis* | C | 104972 | China |
| *O. australiensis* | E | 101410, 105263 | Australia |
| *O. brachyantha* | F | 105151 | Sierra Leone |
| *O. granulata* | G | 106469 | China |
| *Chikusichloa aquatica* | -- | 106186 | Japan |
| *Rhynchoryza subulata* | -- | 100913 | Argentina |
| *Luziola leiocarpa* | -- | 82043 | Argentina |
| *Ehrharta erecta* | -- | 208290 | South Africa |

^a^ All accessions were obtained from the International Rice Research Institute at Los Banos, Philippines.
