# Supplementary material for: Evolutionary rate patterns of the Gibberellin pathway genes
Source: BMC Evol Biol. 2009 Aug 18;9:206. doi: 10.1186/1471-2148-9-206 (PMC2794029; doi:10.1186/1471-2148-9-206)
Supplement: Additional file 4 — table S2. Information of the 7 GA genes sampled in the present study. [file 1471-2148-9-206-S4.doc]

Table 2. Information of the 7 GA genes sampled in the present study.

| Gene | Speices | Length sequenced (bp) | | Coding | | | | Noncoding | Accession  number |
| --- | --- | --- | --- | --- | --- | --- | --- | --- | --- |
|  |  | Total | Coding | ENC ^a^ | GC | GC1,2 ^b^ | GC_3_ | GC |  |
|  | *Oryza sativa* | 1901 | 1002 | 56.16 | 0.460 | 0.443 | 0.494 | 0.329 | AP004572 ^c^ |
|  | *O. punctata* | 1949 | 999 | 56.46 | 0.461 | 0.446 | 0.492 | 0.327 | EF577637 |
|  | *O. officinalis* | 1939 | 1002 | 55.24 | 0.464 | 0.452 | 0.488 | 0.326 | EF577638 |
|  | *O. australiensis* | 1944 | 1002 | 55.91 | 0.456 | 0.448 | 0.474 | 0.316 | EF577639 |
| *CPS1* | *O. brachyantha* | 1906 | 1002 | 56.47 | 0.490 | 0.448 | 0.574 | 0.336 | EF577640 |
|  | *O. granulata* | 1861 | 1002 | 53.82 | 0.458 | 0.442 | 0.491 | 0.311 | EF577641 |
|  | *Luziola leiocarpa* | 2389 | 1002 | 52.03 | 0.459 | 0.442 | 0.494 | 0.296 | EU179433 |
|  | *Chikusichloa aquatica* | 2003 | 1002 | 53.32 | 0.454 | 0.449 | 0.465 | 0.315 | EU179434 |
|  | *Rhynchoryza subulata* | 2361 | 1002 | 53.55 | 0.457 | 0.451 | 0.470 | 0.330 | EU179435 |
|  | *Ehrharta erecta* | 2570 | 1002 | 52.73 | 0.429 | 0.430 | 0.429 | 0.318 | EU179432 |
|  | Mean (SE) ^d^ | 2038±74.99 | 1002±0.00 | 54.56±0.57 | 0.462±0.004 | 0.447±0.001 | 0.494±0.012 | 0.320±0.005 |  |
|  | *O. sativa* | 1844 | 1020 | 55.10 | 0.434 | 0.440 | 0.403 | 0.368 | OSJN00255 ^c^ |
|  | *O. punctata* | 2095 | 1023 | 54.19 | 0.422 | 0.427 | 0.413 | 0.373 | EF577651 |
|  | *O. officinalis* | 1820 | 1020 | 53.64 | 0.427 | 0.434 | 0.414 | 0.373 | EF577652 |
|  | *O. australiensis* | 1829 | 1020 | 54.46 | 0.429 | 0.430 | 0.429 | 0.371 | EF577653 |
| *KS1* | *O. brachyantha* | 1859 | 1023 | 54.77 | 0.427 | 0.428 | 0.425 | 0.357 | EF577654 |
|  | *O. granulata* | 1895 | 1020 | 52.19 | 0.426 | 0.430 | 0.420 | 0.368 | EF577655 |
|  | *Luziola leiocarpa* | 1935 | 1020 | 53.11 | 0.432 | 0.440 | 0.417 | 0.352 | EU179418 |
|  | *Chikusichloa aquatica* | 1899 | 1023 | 49.40 | 0.416 | 0.424 | 0.402 | 0.365 | EU179419 |
|  | *Rhynchoryza subulata* | 1838 | 1023 | 49.79 | 0.414 | 0.421 | 0.402 | 0.368 | EU179420 |
|  | *Ehrharta erecta* | 1872 | 1023 | 53.34 | 0.426 | 0.431 | 0.416 | 0.353 | EU179421 |
|  | Mean (SE) ^d^ | 1864.88±14.32 | 1021.13±0.55 | 52.81±0.78 | 0.426±0.003 | 0.431±0.002 | 0.417±0.004 | 0.365±0.003 |  |
|  | *O. sativa* | 2160 | 1047 | 58.55 | 0.514 | 0.444 | 0.653 | 0.357 | AP005471 ^c^ |
|  | *O. punctata* | 2203 | 1050 | 55.79 | 0.518 | 0.445 | 0.660 | 0.347 | EF577644 |
|  | *O. officinalis* | 2359 | 1047 | 57.39 | 0.522 | 0.452 | 0.665 | 0.378 | EF577645 |
|  | *O. australiensis* | 1846 | 1047 | 56.52 | 0.516 | 0.445 | 0.659 | 0.373 | EF577646 |
| *KO2* | *O. brachyantha* | 2806 | 1047 | 56.73 | 0.526 | 0.461 | 0.656 | 0.328 | EF577647 |
|  | *O. granulata* | 2061 | 1047 | 57.01 | 0.506 | 0.448 | 0.622 | 0.371 | EF577648 |
|  | *Luziola leiocarpa* | 2158 | 1050 | 59.04 | 0.509 | 0.455 | 0.617 | 0.338 | EU179413 |
|  | *Chikusichloa aquatica* | 2074 | 1047 | 61.00 | 0.505 | 0.456 | 0.604 | 0.351 | EU179414 |
|  | *Rhynchoryza subulata* | 2053 | 1047 | 59.41 | 0.516 | 0.457 | 0.633 | 0.364 | EU179415 |
|  | *Ehrharta erecta* | 2000 | 1047 | 58.54 | 0.515 | 0.446 | 0.653 | 0.333 | EU179416 |
|  | Mean (SE) ^d^ | 2189.63±101.52 | 1047.38±0.38 | 58.21±0.55 | 0.514±0.003 | 0.452±0.002 | 0.639±0.008 | 0.358±0.006 |  |
|  | *O. sativa* | 2231 | 1053 | 35.02 | 0.600 | 0.492 | 0.818 | 0.307 | AP000616 ^c^ |
|  | *O. punctata* | 1833 | 1053 | 35.13 | 0.597 | 0.486 | 0.820 | 0.333 | EF577665 |
|  | *O. officinalis* | 1844 | 1053 | 39.10 | 0.600 | 0.493 | 0.815 | 0.328 | EF577666 |
|  | *O. australiensis* | 1867 | 1053 | 39.27 | 0.602 | 0.495 | 0.818 | 0.334 | EF577667 |
| *KAO* | *O. brachyantha* | 2626 | 1053 | 39.20 | 0.606 | 0.498 | 0.823 | 0.334 | EF577668 |
|  | *O. granulata* | 1808 | 1053 | 37.72 | 0.612 | 0.501 | 0.832 | 0.336 | EF577669 |
|  | *Luziola leiocarpa* | 1826 | 1050 | 38.67 | 0.612 | 0.503 | 0.831 | 0.336 | EU179408 |
|  | *Chikusichloa aquatica* | 1772 | 1047 | 42.48 | 0.598 | 0.490 | 0.814 | 0.338 | EU179409 |
|  | *Rhynchoryza subulata* | 1790 | 1047 | 42.02 | 0.595 | 0.490 | 0.805 | 0.328 | EU179410 |
|  | *Ehrharta erecta* | 2363 | 1026 | 53.65 | 0.541 | 0.451 | 0.723 | 0.324 | EU179411 |
|  | Mean (SE) ^d^ | 1970.5±107.20 | 1051.13±0.97 | 39.19±0.83 | 0.603±0.002 | 0.495±0.002 | 0.820±0.003 | 0.330±0.004 |  |
|  | *O. sativa* | 699 | 597 | 29.39 | 0.715 | 0.59 | 0.965 | 0.402 | AP003561 ^c^ |
|  | *O. punctata* | 710 | 597 | 31.54 | 0.702 | 0.583 | 0.940 | 0.443 | EU179376 |
|  | *O. officinalis* | 708 | 597 | 30.03 | 0.714 | 0.585 | 0.970 | 0.496 | EU179377 |
|  | *O. australiensis* | 694 | 597 | 29.73 | 0.714 | 0.585 | 0.970 | 0.598 | EU179378 |
| *GA20ox2* | *O. brachyantha* | 706 | 597 | 30.24 | 0.715 | 0.588 | 0.970 | 0.569 | EU179379 |
|  | *O. granulata* | 695 | 597 | 31.1 | 0.710 | 0.588 | 0.955 | 0.439 | EU179380 |
|  | *Luziola leiocarpa* | 700 | 597 | 29.88 | 0.715 | 0.586 | 0.975 | 0.427 | EU179382 |
|  | *Chikusichloa aquatica* | 691 | 597 | 29.20 | 0.717 | 0.591 | 0.970 | 0.426 | EU179383 |
|  | *Rhynchoryza subulata* | 694 | 597 | 30.79 | 0.709 | 0.586 | 0.955 | 0.495 | EU179384 |
|  | *Ehrharta erecta* | 698 | 597 | 31.67 | 0.702 | 0.575 | 0.955 | 0.475 | EU179385 |
|  | Mean (SE) ^d^ | 698.38±2.15 | 597±0.00 | 30.05±0.23 | 0.714±0.001 | 0.587±0.001 | 0.966±0.003 | 0.482±0.025 |  |
|  | *O. sativa* | 887 | 780 | 31.49 | 0.714 | 0.589 | 0.965 | 0.336 | AP002523 ^c^ |
|  | *O. punctata* | 945 | 780 | 32.07 | 0.700 | 0.573 | 0.954 | 0.467 | EU179398 |
|  | *O. officinalis* | 909 | 780 | 31.60 | 0.701 | 0.576 | 0.954 | 0.442 | EU179399 |
|  | *O. australiensis* | 939 | 780 | 30.17 | 0.710 | 0.583 | 0.965 | 0.459 | EU179400 |
| *GA3ox2* | *O. brachyantha* | 885 | 780 | 28.81 | 0.729 | 0.599 | 0.992 | 0.429 | EU179401 |
|  | *O. granulata* | 910 | 780 | 30.01 | 0.709 | 0.581 | 0.965 | 0.454 | EU179402 |
|  | *Luziola leiocarpa* | 965 | 786 | 30.21 | 0.702 | 0.571 | 0.965 | 0.335 | EU179403 |
|  | *Chikusichloa aquatica* | 943 | 780 | 30.86 | 0.703 | 0.579 | 0.950 | 0.491 | EU179404 |
|  | *Rhynchoryza subulata* | 944 | 780 | 30.03 | 0.709 | 0.583 | 0.961 | 0.470 | EU179405 |
|  | *Ehrharta erecta* | 972 | 780 | 29.68 | 0.713 | 0.577 | 0.985 | 0.453 | EU179406 |
|  | Mean (SE) ^d^ | 922.75±10.32 | 780.75±0.75 | 30.40±0.32 | 0.710±0.003 | 0.583±0.003 | 0.965±0.004 | 0.427±0.021 |  |
|  | *O. sativa* | 885 | 798 | 31.95 | 0.706 | 0.579 | 0.958 | 0.529 | AC132485 ^c^ |
|  | *O. punctata* | 900 | 792 | 35.77 | 0.682 | 0.572 | 0.901 | 0.491 | EU179387 |
|  | *O. officinalis* | 890 | 801 | 34.83 | 0.687 | 0.574 | 0.914 | 0.483 | EU179388 |
|  | *O. australiensis* | 876 | 786 | 34.18 | 0.686 | 0.567 | 0.924 | 0.489 | EU179389 |
| *GA2ox4* | *O. brachyantha* | 1059 | 792 | 33.64 | 0.699 | 0.584 | 0.932 | 0.506 | EU179390 |
|  | *O. granulata* | 974 | 789 | 34.70 | 0.692 | 0.58 | 0.916 | 0.497 | EU179391 |
|  | *Luziola leiocarpa* | 916 | 786 | 34.74 | 0.702 | 0.595 | 0.916 | 0.592 | EU179393 |
|  | *Chikusichloa aquatica* | 997 | 774 | 34.32 | 0.689 | 0.572 | 0.922 | 0.511 | EU179394 |
|  | *Rhynchoryza subulata* | 1005 | 774 | 33.88 | 0.689 | 0.57 | 0.927 | 0.502 | EU179395 |
|  | *Ehrharta erecta* | 861 | 780 | 30.42 | 0.728 | 0.604 | 0.977 | 0.531 | EU179396 |
|  | Mean (SE) ^d^ | 950.25±23.96 | 787.5±3.49 | 34.03±0.33 | 0.694±0.003 | 0.578±0.003 | 0.926±0.005 | 0.514±0.012 |  |

^a^ ENC, effective number of codons (Wright, 1990).

^b^ GC1, 2 is G+C content at first and second codon positions.

^c^ Sequences downloaded from GenBank.

^d^ Average of 8 species of Oryzeae.
