# Supplementary material for: Evolutionary rate patterns of the Gibberellin pathway genes
Source: BMC Evol Biol. 2009 Aug 18;9:206. doi: 10.1186/1471-2148-9-206 (PMC2794029; doi:10.1186/1471-2148-9-206)
Supplement: Additional file 5 — table S3. Sequences and melting temperatures (Tm) of internal primers used for sequencing of four genes that are ~2 kb in length. [file 1471-2148-9-206-S5.doc]

**Table 3**. Sequences and melting temperatures (Tm) of internal primers used for sequencing of four genes that are ~ 2 kb in length.

**CPS1**

1. CPS444F Tm = 57.3℃

CPS444F: 5’- TGGCAGAGGCTTCTGAAG -3’

1. CPS527F Tm = 55.4℃

CPS527F: 5’-CggTgACAAgAAATgCTTCg-3’

1. CPS971F Tm = 59.6℃

CPS971F: 5'-TTGCTGGGCTAGGAACTCC-3'

1. CPS1014F Tm = 60.07℃

CPS1014F: 5’- GCTATGGCTTTCCGTCTACTAC -3’

**KS1**

1. KS515F Tm = 56.1℃

KS515F: 5’-GCTTTGGCTGTTGAAGATTTC-3’

1. KS866F Tm = 49.8℃

KS866F: 5’-CCTCATTGCTTTAGTTG-3’

1. KS1419F Tm = 53.7℃

KS1419F: 5’- TTTAGACTGATGAGCACTTG -3’

1. KS1443R Tm = 57.3℃

KS1443R: 5’-GCTGTCATTCAGGAGACG-3’

**KO2**

1. KO124F Tm = 53.2℃

KO124F: 5’- CTGTAGTTGTGCTCAATTC - 3’

1. KO2289R Tm = 57.9℃

KO2289R: 5’-GCCATCGTCTTGTACATGTC - 3’

1. KO2391R Tm = 59.7℃

KO2391R: 5’-TCAGCCTCCACYCGAACTC - 3’

**KAO**

1. KAO707F Tm = 61.9℃

KAO707F: 5’- ACCGTCTTCCTCCAGGAGAAC-3’

1. KAO931F Tm = 57.6℃

KAO931F: 5’- GATGCACTTCCTCTCACAG -3’

1. KAO1478F Tm = 60℃

KAO1478F: 5’- CGTCAACATCTCCTTCGTGTC -3’

1. KAOF Tm = 54.59℃

KAOF: 5’- CGCAAGAAGCTAGTGTC-3’
