# Supplementary material for: Evolutionary rate patterns of the Gibberellin pathway genes
Source: BMC Evol Biol. 2009 Aug 18;9:206. doi: 10.1186/1471-2148-9-206 (PMC2794029; doi:10.1186/1471-2148-9-206)
Supplement: Additional file 6 — figure S3. Pruned phylogeny tree consisting of 6 species. Branches b1 to b10 are 10 independent branches. [file 1471-2148-9-206-S6.doc]

*O.officinalis*

*O.australiensis*

*O.granulata*

*C.aquatica*

*R.subulata*

*L.leiocarpa*

b1

b2

b3

b4

b5

b6

b7

b9

b8

b10

**Figure 3**. Pruned phylogeny tree consisting of 6 species. Branches b1 to b10 are 10 independent branches.
