# Supplementary material for: Evolutionary rate patterns of the Gibberellin pathway genes
Source: BMC Evol Biol. 2009 Aug 18;9:206. doi: 10.1186/1471-2148-9-206 (PMC2794029; doi:10.1186/1471-2148-9-206)
Supplement: Additional file 7 — table S4. Wilcoxon signed rank test of pairwise comparisons of dN and dS between genes for 10 independent branches of the pruned tree consisting of 6 species. [file 1471-2148-9-206-S7.doc]

**Table 4** Wilcoxon signed rank test of pairwise comparisons of *d*_N_ and *d*_S_ between genes for 10 independent branches of the pruned tree consisting of 6 species.

| Comparison | *d*_S_ | | | | *d*_N_ | | | |
| --- | --- | --- | --- | --- | --- | --- | --- | --- |
| Gene1 vs. Gene2 | Gene1>Gene2 | Gene1<Gene2 | Gene1=Gene2 | P | Gene1>Gene2 | Gene1<Gene2 | Gene1=Gene2 | P |
| *KS1* vs. *CPS1* | 2 | 7 | 1 | **0.055** | 10 | 0 | 0 | 0.002 |
| *KO2* vs. *CPS1* | 4 | 5 | 1 | **0.203** | 8 | 2 | 0 | 0.020 |
| *KO2* vs. *KS1* | 6 | 3 | 1 | **0.250** | 1 | 9 | 0 | 0.004 |
| *KAO* vs. *CPS1* | 7 | 2 | 1 | 0.020 | 5 | 5 | 0 | **0.695** |
| *KAO* vs. *KS1* | 8 | 1 | 1 | 0.008 | 1 | 9 | 0 | 0.004 |
| *KAO* vs. *KO2* | 8 | 1 | 1 | 0.008 | 2 | 8 | 0 | 0.232 |
| *GA20ox2* vs. *CPS1* | 9 | 1 | 0 | 0.006 | 0 | 10 | 0 | 0.002 |
| *GA20ox2* vs. *KS1* | 9 | 1 | 0 | 0.004 | 0 | 10 | 0 | 0.002 |
| *GA20ox2* vs. *KO2* | 9 | 1 | 0 | 0.004 | 0 | 10 | 0 | 0.002 |
| *GA20ox2* vs. *KAO* | 7 | 2 | 1 | **0.203** | 1 | 9 | 0 | 0.004 |
| *GA3ox2* vs. *CPS1* | 9 | 1 | 0 | 0.006 | 6 | 4 | 0 | **0.557** |
| *GA3ox2* vs. *KS1* | 9 | 1 | 0 | 0.004 | 2 | 8 | 0 | 0.020 |
| *GA3ox2* vs. *KO2* | 9 | 1 | 0 | 0.004 | 3 | 7 | 0 | **0.770** |
| *GA3ox2* vs. *KAO* | 7 | 2 | 1 | **0.129** | 6 | 4 | 0 | **0.846** |
| *GA3ox2* vs. *GA20ox2* | 4 | 5 | 1 | **0.910** | 9 | 1 | 0 | 0.004 |
| *GA2ox4* vs. *CPS1* | 8 | 1 | 1 | 0.012 | 10 | 0 | 0 | 0.002 |
| *GA2ox4* vs. *KS1* | 8 | 1 | 1 | 0.008 | 7 | 3 | 0 | **0.322** |
| *GA2ox4* vs. *KO2* | 8 | 1 | 1 | 0.008 | 9 | 1 | 0 | 0.014 |
| *GA2ox4* vs. *KAO* | 5 | 3 | 2 | **0.547** | 9 | 1 | 0 | 0.004 |
| *GA2ox4* vs. *GA20ox2* | 3 | 6 | 1 | **0.570** | 10 | 0 | 0 | 0.002 |
| *GA2ox4* vs. *GA3ox2* | 4 | 5 | 1 | **0.570** | 9 | 1 | 0 | 0.006 |
