# Supplementary material for: Evolutionary rate patterns of the Gibberellin pathway genes
Source: BMC Evol Biol. 2009 Aug 18;9:206. doi: 10.1186/1471-2148-9-206 (PMC2794029; doi:10.1186/1471-2148-9-206)
Supplement: Additional file 8 — table S5. Log-likelihood values, ω ratios and parameter estimates under models of variable ω ratios among codon sites. [file 1471-2148-9-206-S8.doc]

**Table 5**. Log-likelihood values, ω ratios and parameter estimates under models of variable ω ratios among codon sites

| Gene | Model | p ^a^ | lnL ^b^ | Estimates of Parameters ^c^ | Models Compared | 2ΔL | *P*-value |
| --- | --- | --- | --- | --- | --- | --- | --- |
|  | Mf | 31 | -3022.81 | ω = 0.0001 ~ 0.635, tree length = 1.137, kappa(ts/tv) = 2.257 | M0-Mf | 22.82 | ＞0.05 |
|  | M0 | 17 | -3034.22 | ω = 0.144, tree length ^d^ = 1.131, kappa(ts/tv) = 2.239 |
|  | M1a | 18 | -3000.63 | ω_0_ = 0.054, (p_0_ =0.889); ω_1_ = 1.000, (p_1_ = 0.111) | M1a-M2a | 0 | 1 |
| *CPS1* | M2a | 20 | -3000.63 | p_0_ = 0.889, p_1_ = 0.111, p_2_ = 0.000, ω_2_ = 26.533 |
|  | M7 | 18 | -3001.48 | p = 0.142, q = 0.794 | M7-M8 | 2.28 | ＞0.25 |
|  | M8 | 20 | -3000.34 | p_0_ = 0.923, p = 0.430, q = 5.060; (p_1_= 0.077) ω_1_ = 1.117 |
|  | Mf | 31 | -3332.81 | ω =0.156 ~ 0.849, tree length = 1.234, kappa(ts/tv) = 3.606 | M0-Mf | 16.7 | ＞0.25 |
|  | M0 | 17 | -3341.16 | ω = 0.386, tree length = 1.234, kappa(ts/tv) = 3.608 |
|  | M1a | 18 | -3305.70 | ω_0_ = 0.074, (p_0_ = 0.656); ω_1_ = 1.000, (p_1_ = 0.344) | M1a-M2a | 3.72 | ＞0.1 |
| *KS1* | M2a | 20 | -3303.84 | p_0_ = 0.656, p_1_ = 0.330, p_2_ = 0.014, ω_2_ = 3.753 |
|  | M7 | 18 | -3306.22 | p = 0.135, q = 0.213 | M7-M8 | 5.3 | ＞0.05 |
|  | M8 | 20 | -3303.57 | p_0_ = 0.973, p = 0.205, q = 0.370; (p_1_= 0.027) ω_1_ = 2.994 |
|  | Mf | 31 | -3111.95 | ω = 0.0001 ~ 0.930, tree length = 1.037, kappa(ts/tv) = 2.128 | M0-Mf | 12.98 | ＞0.5 |
|  | M0 | 17 | -3118.44 | ω = 0.177, tree length = 1.033, kappa (ts/tv) = 2.126 |
|  | M1a | 18 | -3093.17 | ω_0_ = 0.076, (p_0_ = 0.864); ω_1_ = 1.000, (p_1_ = 0.136) | M1a-M2a | 0 | 1 |
| *KO2* | M2a | 20 | -3093.17 | p_0_ = 0.864, p_1_ = 0.136, p_2_ = 0.000, ω_2_ = 2.820 |
|  | M7 | 18 | -3093.53 | p = 0.213, q = 0.890 | M7-M8 | 1.48 | ＞0.25 |
|  | M8 | 20 | -3092.79 | p_0_ = 0.963, p = 0.341, q = 1.925; (p_1_= 0.037) ω_1_ = 1.474 |
|  | Mf | 31 | -3063.21 | ω = 0.0001 ~ 813.635, tree length = 1.132, kappa(ts/tv) = 0.948 | M0-Mf | 17.56 | ＞0.1 |
|  | M0 | 17 | -3071.99 | ω = 0.074, tree length = 1.133, kappa (ts/tv) = 0.936 |
|  | M1a | 18 | -3034.15 | ω_0_ = 0.037, (p_0_ = 0.925); ω_1_ = 1.000 (p_1_ = 0.075) | M1a-M2a | 0 | 1 |
| *KAO* | M2a | 20 | -3034.15 | p_0_ = 0.925, p_1_ = 0.075, p_2_ = 0.000, ω_2_ = 26.337 |
|  | M7 | 18 | -3025.22 | p = 0.138, q = 1.417 | M7-M8 | 0 | 1 |
|  | M8 | 20 | -3025.22 | p_0_ = 0.996, p = 0.141, q = 1.521; (p_1_= 0.004) ω_1_ = 1.000 |
|  | Mf | 31 | -1364.50 | ω =0.0001 ~ 999.000, tree length = 0.773, kappa(ts/tv) = 2.388 | M0-Mf | 17.32 | ＞0.1 |
|  | M0 | 17 | -1373.16 | ω = 0.022, tree length = 0.768, kappa (ts/tv) = 2.423 |
|  | M1a | 18 | -1368.60 | ω_0_ = 0.018, (p_0_ = 0.991); ω_1_ = 1.000 (p_1_ = 0.009) | M1a-M2a | 0 | 1 |
| *GA20ox2* | M2a | 20 | -1368.60 | p_0_ = 0.991, p_1_ = 0.009, p_2_ = 0.000, ω_2_ = 42.007 |
|  | M7 | 18 | -1365.07 | p = 0.095, q = 3.262 | M7-M8 | 0 | 1 |
|  | M8 | 20 | -1365.07 | p_0_ = 1.000, p = 0.095, q = 3.262; (p_1_= 0.000) ω_1_ = 1.826 |
|  | Mf | 31 | -2207.21 | ω =0.033 ~ 999.000, tree length = 1.186, kappa(ts/tv) = 2.177 | M0-Mf | 6.5 | ＞0.95 |
|  | M0 | 17 | -2210.46 | ω = 0.073, tree length = 1.202, kappa (ts/tv) = 2.222 |
|  | M1a | 18 | -2173.63 | ω_0_ = 0.038, (p_0_ = 0.920); ω_1_ = 1.000 (p_1_ = 0.080) | M1a-M2a | 0 | 1 |
| *GA3ox2* | M2a | 20 | -2173.63 | p_0_ = 0.920, p_1_ = 0.080, p_2_ = 0.000, ω_2_ = 18.621 |
|  | M7 | 18 | -2166.90 | p = 0.144, q = 1.450 | M7-M8 | 0.88 | ＞0.5 |
|  | M8 | 20 | -2166.46 | p_0_ = 0.980, p = 0.174, q = 2.244; (p_1_= 0.020) ω_1_ = 1.042 |
|  | Mf | 31 | -2723.13 | ω =0.0001 ~ 999.000, tree length = 1.604, kappa(ts/tv) = 2.695 | M0-Mf | 19.84 | ＞0.1 |
|  | M0 | 17 | -2733.05 | ω = 0.129, tree length = 1.621, kappa (ts/tv) = 2.726 |
|  | M1a | 18 | -2699.36 | ω_0_ = 0.074, (p_0_ = 0.877); ω_1_ = 1.000, (p_1_ = 0.123) | M1a-M2a | 0 | 1 |
| *GA2ox4* | M2a | 20 | -2699.36 | p_0_ = 0.877, p_1_ = 0.090, p_2_ = 0.033, ω_2_ = 1.000 |
|  | M7 | 18 | -2694.51 | p = 0.326, q = 1.872 | M7-M8 | 0.92 | ＞0.5 |
|  | M8 | 20 | -2694.05 | p_0_ = 0.963, p = 0.414, q = 3.011; (p_1_= 0.037) ω_1_ = 1.000 |

a p, number of parameters.

^b^ Log-likelihood values of the data given the model.

^c^ Estimates of parameters under different models.

^d^ Tree length is the sum of branch length.
